# Neutral Sphingomyelinase Behaviour in Hippocampus Neuroinflammation of MPTP-Induced Mouse Model of Parkinson's Disease and in Embryonic Hippocampal Cells

**DOI:** 10.1155/2017/2470950

**Published:** 2017-11-16

**Authors:** Samuela Cataldi, Cataldo Arcuri, Stéphane Hunot, François-Pierre Légeron, Carmen Mecca, Mercedes Garcia-Gil, Andrea Lazzarini, Michela Codini, Tommaso Beccari, Anna Tasegian, Bernard Fioretti, Giovanna Traina, Francesco Saverio Ambesi-Impiombato, Francesco Curcio, Elisabetta Albi

**Affiliations:** ^1^Department of Pharmaceutical Science, University of Perugia, Perugia, Italy; ^2^Department of Experimental Medicine, University of Perugia, Polo Unico Sant'Andrea delle Fratte, Piazzale Gambuli, 06132 Perugia, Italy; ^3^Inserm U 1127, CNRS UMR 7225, Sorbonne Universités, UPMC Univ Paris 06 UMR S 1127, Institut du Cerveau et de la Moelleépinière (ICM), 75013 Paris, France; ^4^Department of Biology and Interdepartmental Research Center Nutrafood “Nutraceuticals and Food for Health”, University of Pisa, Pisa, Italy; ^5^CRABiON-Perugia, Via Ponchielli 4, 06073 Perugia, Italy; ^6^Department of Chemistry, Biology and Biotechnology, Via Elce di sotto, 06123 Perugia, Italy; ^7^Dipartimento di Area Medica (DAME), University of Udine, P.le M. Kolbe 4, 33100 Udine, Italy

## Abstract

Neutral sphingomyelinase is known to be implicated in growth arrest, differentiation, proliferation, and apoptosis. Although previous studies have reported the involvement of neutral sphingomyelinase in hippocampus physiopathology, its behavior in the hippocampus during Parkinson's disease remains undetected. In this study, we show an upregulation of inducible nitric oxide synthase and a downregulation of neutral sphingomyelinase in the hippocampus of 1-methyl-4-phenyl-1,2,3,6-tetrahydropyridine- (MPTP-) induced mouse model of Parkinson's disease. Moreover, the stimulation of neutral sphingomyelinase activity with vitamin 1,25-dihydroxyvitamin D3 reduces specifically saturated fatty acid sphingomyelin by making sphingomyelin a less rigid molecule that might influence neurite plasticity. The possible biological relevance of the increase of neutral sphingomyelinase in Parkinson's disease is discussed.

## 1. Introduction

Neuroinflammation was described to be involved in the pathogenesis of Parkinson's disease [1]. It has been previously demonstrated that in 1-methyl-4-phenyl-1,2,3,6-tetrahydropyridine- (MPTP-) induced mouse model of Parkinson's disease (PD), the midbrain is characterized by an overexpression of e-cadherin and interleukin-6 accompanied by a reduction of tyrosine hydroxylase for dopamine synthesis [2]. On the other hand, in MPTP-induced Parkinson, the increase in proinflammatory cytokines was attenuated by using FK506, an immunosuppressant drug [3]. Castro-Hernández et al. demonstrated that MPTP-induced dopaminergic damage had time-dependent effects; the neurotoxic effect was evident mainly in the ventral region of the hippocampus [4]. Recently, an age-dependent hippocampal volume loss with cognitive impairment was shown in patients with PD [5]. Lipopolysaccharide-induced neuroinflammation changed the lipid composition of hippocampal plasma membranes from rats [6], and modulation of neuroinflammation by sphingolipids has been described [7]. Glucocorticosterone induced the release of ceramide into the extracellular space of the hippocampus, by reducing neuronal stem cell proliferation [8]. Ceramides are a class of sphingolipid, produced by sphingomyenase (SMase) and de novo synthesis that regulate various cell functions such as proliferation, differentiation, senescence, apoptosis, autophagy, migration, and intracellular trafficking [9]. Neutral SMase (nSMase)/ceramide pathway was described to be involved in hippocampus inflammation during ischemia-associated neuronal damage [10]. The inhibition of nSMase activity reduced ceramide accumulation in astrocytes and alleviated neuronal damage [10]. Moreover, the SMase inhibited the ligand binding function of serotonin1A receptors [11] and reduced M1 muscarinic receptors [12] in the hippocampus. The hydrolysis of sphingomyelin (SM) with production of sphingosine-1-phosphate increased hippocampal neuron excitability [13]. So far, there are no data on nSMAse in the hippocampal neuroinflammation in PD. Here, we have investigated the possible variation of nSMase in relation to the inducible nitric oxide synthase (iNOS) in the hippocampal dentate gyrus of mice with MPTP-induced PD. Our results showed reduction of nSMase. As in the adult dentate gyrus neurogenesis occurs [14], we used 1,25-dihydroxyvitamin D3 (VD3) that induces cell differentiation via nSMase [15, 16] to stimulate nSMase in embryonic hippocampus cells. Then, we studied the variations of SM species in order to understand the possible importance of the reduction of nSMase in PD.

## 2. Methods

### 2.1. Animals

Ten- to twelve-week-old male C57BL/6J mice weighing 25–30 g (CERJ, France) were used as previously reported [2]. Mice were kept in a temperature-controlled room (23°C ± 1°C) under a 12-hour light/dark cycle with access to food and water ad libitum. Animal treatments were performed according to ethical regulations and guidelines (Guide for the Care and Use of Laboratory Animals, NIH publication number 85-23, revised 1985) and the European Communities Council Directive 86/609/EEC. Experimental protocols were performed following the French national chart for ethics of animal experiments (articles R 214-87 to 126 of the “Code rural”) and received approval from the ethical committee number 5 “Charles Darwin” and from the ICM animal care and use committee.

### 2.2. Reagents

Anti-nSMase and anti-NOS2 (M-19) were from Santa Cruz Biotechnology Inc. (California, USA). SDS-PAGE molecular weight standard was from Bio-Rad Laboratories (Hercules, CA, USA). VD3 was obtained from DBA Italia (Segrate, Milan, Italy). Dulbecco's modified Eagle's medium (DMEM), bovine serum albumin, tetramethylrhodamineisothiocyanate-conjugated goat anti-rabbit IgG, and MPTP-HCl were from Sigma Chemical Co. (St. Louis, Missouri, USA). Lipid standards 16:0SM, 18:1SM, and 24:0SM were purchased from Avanti (Avanti Polar, Alabaster, AL, USA).

### 2.3. MPTP Injection and Tissue Preparation

Animals were treated as previously reported [2]. Groups of mice (*n* = 5) received MPTP under an acute protocol. Mice were given 4 i.p. injections of MPTP-HCl 2 hours apart and at a dose of 20 mg/kg (free-base). They were euthanized 7 days after the last MPTP injection. Control mice received an equivalent volume of 0.9% NaCl solution. Removed brains were postfixed overnight in fresh 4% paraformaldehyde (PFA)/phosphate-buffered saline (PBS) solution, cryoprotected with 30% sucrose in 0.1 M PB and frozen in isopentane (−30°C). Brain free-floating sections (20 *μ*m thick) encompassing the hippocampus were prepared using a freezing microtome (Microm, Germany). Sections were stored at −70°C until use.

### 2.4. Immunofluorescence

The cryostat sections were incubated overnight with 3% (*w*/*v*) bovine serum albumin (BSA) and 1% (*w*/*v*) glycine in PBS to block nonspecific sites as previously reported [17]. Then, sections were incubated with anti-iNOS or anti-nSMase primary antibodies diluted 1 : 100 in 3% (*w*/*v*) BSA in PBS for 1 hr, washed three times in 0.1% (*v*/*v*) Tween-20 in PBS and two times in PBS, incubated with tetramethylrhodamineisothiocyanate-conjugated anti-rabbit IgG for 1 h, diluted 1 : 50 in 3% (*w*/*v*) BSA in PBS, and washed as above. The samples were mounted in 80% (*w*/*v*) glycerol, containing 0.02% (*w*/*v*) NaN_3_ and p-phenylenediamine (1 mg/ml) in PBS to prevent fluorescence fading. The antibody incubations were done in a humid chamber at room temperature. Fluorescent analysis was performed on a DMRB Leica epi-fluorescent microscope equipped with a digital camera.

### 2.5. Cell Culture and Treatments

Immortalized hippocampal neurons HN9.10e (kind gift of Dr. Kieran Breen, Ninewells Hospital, Dundee, UK) were cultured as previously reported [16]. VD3, dissolved in absolute ethanol as a vehicle at the 100 nM physiological concentration, was added to the cultures for 48 h; in control samples, only absolute ethanol was added [16]. The cells were used in part for the analysis of total protein content, in part for enzyme activity assay, and in part for lipidomic analysis of SM.

### 2.6. Protein Content

Total protein concentration was evaluated spectrophotometrically at 750 nm by using albumin bovine serum as a standard, as previously reported [18].

### 2.7. Enzyme Activity Assay

Enzymes involved in nervous cell differentiation (nSMase) [16] and lysosomal enzymes involved in nervous cell damage such as acid sphingomyelinase (aSMase), *β*-hexosaminidase, *α*-fucosidase, *β*-mannosidase, *α*-mannosidase, *β*-galactosidase, and *β*-glucocerebrosidase [19, 20] were assayed. The nSMase activity was measured as previously reported [21]. Briefly, ^14^C–SM (final specific activity 1.08 Ci/mol) was used as substrate. The reaction mixture contained 0.1 M Tris/HCl pH 7.6, 0.1 mM ^14^C–SM, 6 mM MgCl_2_, 0.1% Triton X-100, and 100 *μ*g protein to a final volume of 0.1 ml. Incubations were performed at 37°C for 45 min. 2 ml chloroform and methanol (2 : 1) were used to stop the reaction, and 0.4 ml of 0.5% NaCl was added. Samples were centrifuged at 2000 rpm 10 min, and 0.5 ml of the upper phase was diluted in counting vials with 10 ml Ecoscint A and 1 ml distilled water; radioactivity was measured with a Packard liquid scintillation analyzer. The aSMase activity was measured as previously reported [22]. Briefly, the substrate was prepared as nSMase. The reaction mixture contained 1 mM EDTA, pH 5.0, 0.1 mM ^14^C–SM, 250 mM sodium acetate, 0.1% Triton X-100, and 100 *μ*g protein of NFL or nuclei to a final volume of 0.1 ml. Conditions of incubation and radioactive analysis were performed as nSMase. The *β*-hexosaminidase, *α*-fucosidase, *β*-mannosidase, *α*-mannosidase, *β*-galactosidase, and *β*-glucocerebrosidase enzymatic activities were determined with the corresponding fluorogenic substrate and reaction buffer, as previously reported [20]. The fluorescence was measured on a BMG Labtech FLUOstar OPTIMA fluorometer (excitation wavelength  =  360 nm; emission wavelength  =  446 nm).

### 2.8. Ultrafast Liquid Chromatography Tandem Mass Spectrometry (UFLC-MS/MS)

Lipids were extracted, and 16:0SM, 18:1SM, and 24:0SM standards were prepared as previously reported [23]. Analysis was carried out by using ultrafast liquid chromatography system tandem mass spectrometer Applied Biosystem (Shimadzu Italy s.r.l., Italy), and 16:0SM, 18:1SM, and 24:0SM species were analyzed by using external calibrators and identified as previously reported [23]. All SM species were analyzed by identifying the peaks on the basis of their molecular weight by using standards as references [24].

### 2.9. Statistical Analysis

Three experiments were performed for each analysis. Data are expressed as mean ± S.D., and *t-*test was used for statistical analysis between control and experimental samples.

## 3. Results and Discussion

### 3.1. Results

**Figure 1:**
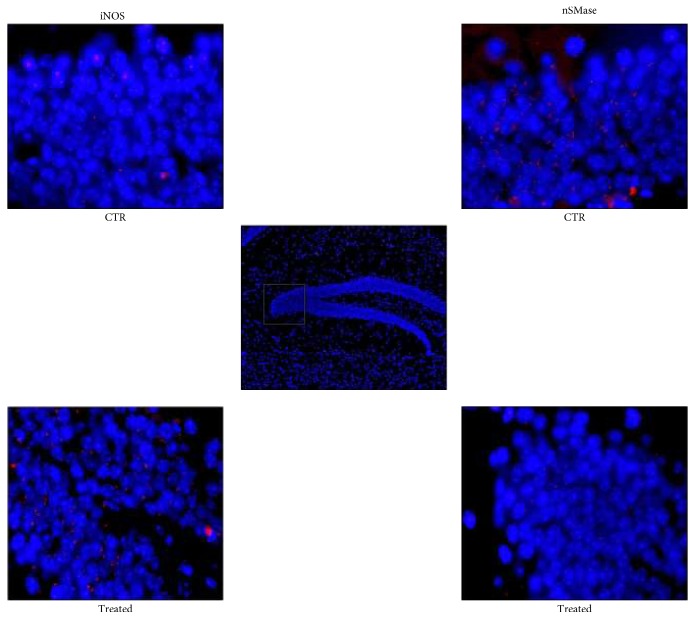
Hippocampus of untreated (control) or MPTP-treated (experimental) mice. The samples were treated as reported in Material and Methods. (c) DAPI fluorescence image of the dentate gyrus of the hippocampus (10x magnification); in the square, detail enlarged in the images of CTR (control) and Treated (experimental). (a, d) iNOS and (b, e) nSMase (100x magnification oil immersion) immunofluorescence. The images represent the merged signals with DAPI signals (blue) in the nuclei and immunolabelling with anti-iNOS or anti-nSMase (red).

**Figure 2:**
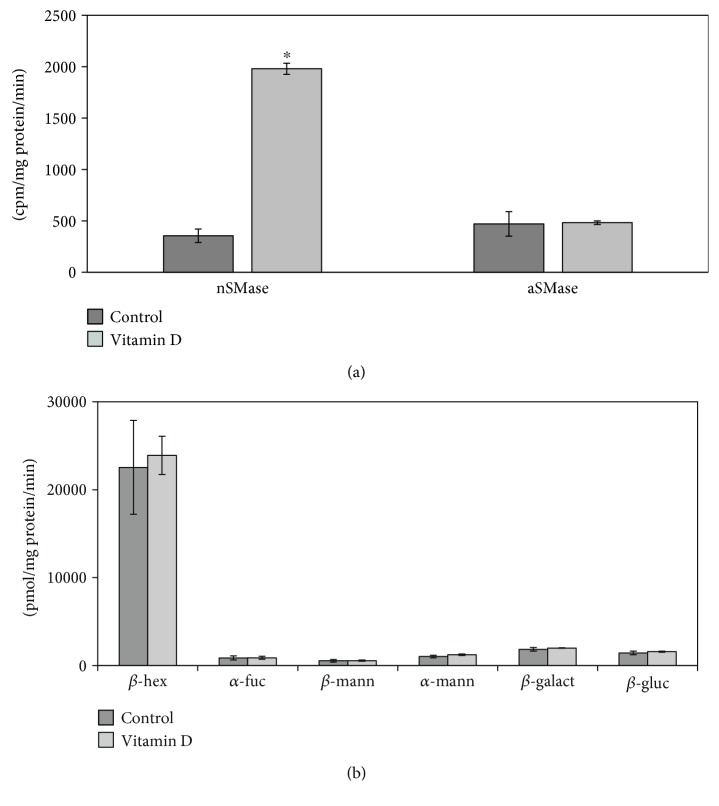
HN9.10 embryonic hippocampal cells cultured in the presence or the absence of VD3 for 48 h. (a) nSMase and aSMase activity; (b) *β*-hexosaminidase (*β*-hex), *α*-fucosidase (*α*-fuc), *β*-mannosidase (*β*-mann), *α*-mannosidase (*α*-mann), *β*-galactosidase (*β*-galact), and *β*-glucocerebrosidase (*β*-gluc) activities. Data are expressed as the mean ± S.D. of 3 independent experiments performed in duplicate. ^∗^*P* < 0.001 versus the control sample.

**Figure 3:**
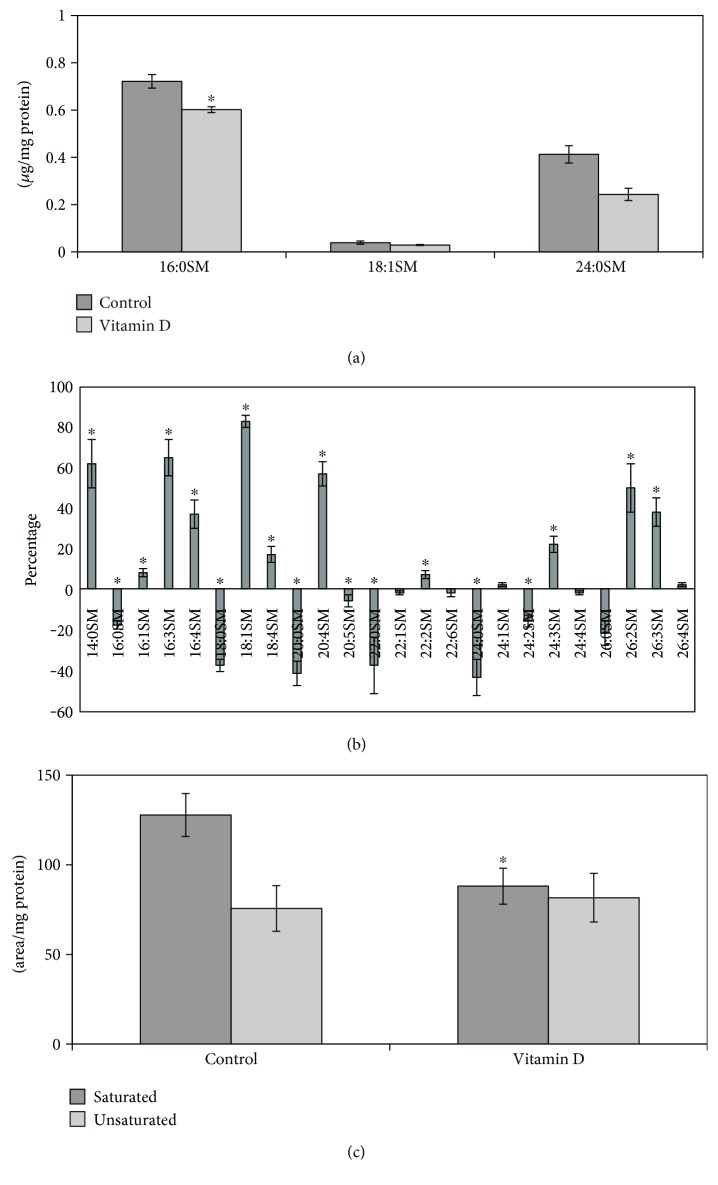
Sphingomyelin in control and vitamin D3-treated HN9.10 cells after 48 h of culture. (a) SM species studied by using 16:0SM, 18:1SM, and 24:0SM external calibrators. Data are expressed as nmol/mg protein and represent the mean ± S.D. of three separated experiments. (b) SM species studied by evaluating the areas of all the peaks identified on the basis of their molecular weight. Data are expressed as area/mg protein and represent the mean ± S.D. of three separated experiments. (c) Total saturated and unsaturated fatty acids. Data are expressed as area/mg protein and represent the mean ± S.D. of three separated experiments. ^∗^*P* < 0.001 versus control sample. (1) 14:0SM; (2) 16:0SM; (3) 16:1SM; (4) 16:3SM; (5) 16:4SM; (6) 18:0SM; (7) 18:1SM; (8) 18:4SM; (9) 20:0SM; (10) 20:4SM; (11) 20:5SM; (12) 22:0SM; (13) 22:1SM; (14) 22:2SM; (15) 22:6SM; (16) 24:0SM; (17) 24:1SM; (18) 24:2SM; (19) 24:3SM; (20) 24:4SM; (21) 26:0SM; (22) 26:2SM; (23) 26:3SM; (24) 26:4SM.

iNOS inflammatory cytokine has been reported to be highly expressed in striatum and substantia nigra of animals with PD [25]. As the hippocampus is damaged during PD [4, 5], we analyzed the difference of iNOS expression in sections of hippocampus from normal and MPTP-induced Parkinson mice. We showed dentate gyrus by using DAPI signal (blue) in the nuclei, and we focused the attention on the corner region (Figure 1). Immunofluorescence staining with the anti-iNOS antibody (red) and merged with DAPI (blue) revealed the higher expression of iNOS in experimental than in control animals (Figure 1). Since the iNOS function in inflammation is well established, our results confirmed the neuroinflammation in the dentate gyrus of the hippocampus from PD mice. In order to determine the relative contribution of nSMase during neuroinflammation in PD, the expression of nSMase protein was measured in the same samples. The merged image with DAPI signals (blue) in the nuclei and nSMase (red) showed a strong reduction of nSMase in experimental sample. Consistent with a significant role for SM in the brain [26], as SMase is the enzyme that catalyzes SM hydrolysis, these data indicated that nSMase could have a role in the dentate gyrus function. At this moment, we do not have data about the activity of nSMase in the hippocampus of the control and PD-induced mice. Future studies will clarify this point. Therefore, we then wondered what was the effect of nSMase stimulation on the SM of hippocampal cells. For this purpose, we conducted an in vitro study by using VD3 that both stimulates nSMase activity and induces differentiation of HN9.10e embryonic hippocampus cells [15, 16]. It would be very useful in the future to mimic the *in vivo* situation of PD mice by using HN9.10e cells after having silenced nSMase expression. Physiological doses of VD3 [16] significantly increased nSMase activity following treatment for 48 h (Figure 2(a)). Then, we wanted to determine if the elevations in enzymatic activity were specific for nSMase or if it was a general response of the enzymes important for the brain function regulation. Thus, enzyme activities of aSMase, *β*-hexosaminidase, *α*-fucosidase, *β*-mannosidase, *α*-mannosidase, *β*-galactosidase, and *β*-glucocerebrosidase were quantified in untreated and VD3-treated HN9.10e cells. The activity of all these enzymes remained unchanged (Figures 2(a) and 2(b)). The experimental results revealed that nSMase was specifically stimulated by VD3. Thus, we hypothesized that its increase in activity could change the SM profile. To test this hypothesis, we performed the analysis of SM species by UFLC-MS/MS. The results showed that VD3 decreased 1.2-fold the amount of 16:0SM, and 1.70-fold that of 24:0SM (Figure 3(a)). To have a deeper insight in SM species containing saturated or unsaturated FAs, we evaluated the areas of all the peaks identified on the basis of their molecular weight and analyzed their values in relation to protein content. Significant decrease in the levels of saturated molecular species was found for 16:0SM, 18:0SM, 20:0SM, 22:0SM, 24:0SM, and 26:0SM, and significant increase was found for unsaturated molecular species 16:3SM, 16:4SM, 18:1SM, 20:4SM, 24:3SM, 26:2SM, and 26:3SM (Figure 3(b)). Then we compared the changes in the total levels of SM species containing saturated and unsaturated FAs. As reported in Figure 3(c), the SM saturated FAs decreased and SM unsaturated FA increased in VD3-treated cells compared with control cells.

### 3.2. Discussion

SMase is an indispensable enzyme for the brain and plays an important role in signal transduction pathways by regulating the level of SM and ceramide species involved in physiological functions and pathological diseases. A wide variety of studies has noted the diversity of nSMase and aSMase functions during brain development/stem cell differentiation and ischemic/degenerative/stress responses [26, 27]. Recent evidence has highlighted the role of nSMase in the physiopathology of the hippocampus [10–12]. The involvement of the hippocampus in PD has been recently described [4, 5]. Yao et al. suggested that hippocampal pathology makes a key contribution to visual hallucinations in PD [28]. How hippocampus is damaged in PD remains unclear. As we know that iNOS is induced by inflammatory cytokines [25], we hypothesized a neuroinflammation in the hippocampus of PD mice. At this point, the possible role of the nSMase downregulation in neuroinflammation remains obscure. Until now, little is known about the specific nSMase role on hippocampus health and/or disease. Gu et al. demonstrated a considerable production of ceramide in astrocytes during early cerebral ischemia accompanied by the induction of nSMase but not aSMase in the rat hippocampi, with ceramide accumulation [29]. Also, Babenko and Shakhova reported that the nSMase, but not the aSMase, increased in both the hippocampus and brain cortex during aging, suggesting that redox-sensitive nSMase played an important role in SM turnover dysregulation in both the hippocampus and neocortex at old age [30]. The authors thus indicated the increase of nSMase as an adverse event. However, Norman et al. highlighted that nSMase increased action potential frequency in hippocampal neurons with a rapid increase in the levels of ceramides and S1P indicating the positive regulation of the excitability of hippocampal neurons via nSMase [13]. Thus, at this point, the work on the topic is scarce and the positive or negative role of the nSMase in the different experimental conditions is discordant. Interestingly, we used VD3 to stimulate nSMase activity in HN9.10e embryonic hippocampus cultured cells in order to study the specific role of this enzyme in the hippocampus. The lack of involvement of aSMase, *β*-hexosaminidase, *α*-fucosidase, *β*-mannosidase, *α*-mannosidase, *β*-galactosidase, and *β*-glucocerebrosidase in the VD3 response was consistent with observations suggesting the specificity of nSMase role in the hippocampus [13, 29, 30]. Thus, previous studies were in line with our findings and therefore we have considered a suitable experimental model to use VD3 to potentiate the specific activity of nSMase and consequently to study the specific effects on SM species of HN9.10e.

Notably, our results indicate that the increase of nSMase activity reduces specifically saturated fatty acids and consequently reduces the ratio saturated/unsaturated fatty acids of SM. So we think that the mechanism underlying the increase in unsaturated fatty acids is not dependent on a transformation of unsaturated to saturated fatty acids but on a specific action of nSMase on saturated SM. Since unsaturated fatty acids make the membrane more fluid [31], we hypothesized that the changes of SM species might determine enhanced dynamic properties of the cells that are induced to differentiation by VD3 [32]. Manduca et al. demonstrated that the inhibition of endocannabinoid 2-arachidonylglycerol (omega 6 fatty acid) degradation is important for synaptic plasticity in n-3 polyunsaturated fatty acid-deficient mice [33]. On the other hand, a diet rich in polyunsaturated fatty acids in mice from the first day of gestation improves plasticity in the brain of mice at postnatal day [34]. Thus, another novel finding of our study is that the reduction of saturated/unsaturated fatty acid ratio induced by nSMase, by making SM a less rigid molecule, might influence neurite plasticity. These data would predict that nSMase stimulation could be useful for the health of hippocampus in PD.

## 4. Conclusions

In conclusion, for the first time, we demonstrate that in the hippocampus from PD-induced mice, the nSMase is strongly reduced in association with neuroinflammation and that the increase in nSMase activity following VD3 treatment in HN9.10e cells decreases specifically only saturated SM. Collectively, these results place nSMase as an essential enzyme for hippocampus function. Further investigation of the potential roles of nSMase in PD could provide a better understanding of the biological relevance of its level and activation in the hippocampus as well as the potential benefits of targeting nSMase with VD3.
